# A Comparative Analysis of Complication Rates in Arthroscopic Repair of the Lateral Ankle Ligament and the Brostrom-Gould Technique: A Systematic Review

**DOI:** 10.7759/cureus.48460

**Published:** 2023-11-07

**Authors:** Ali Alhaddad, Amin G Gronfula, Thamer H Alsharif, Ahmed Khawjah, Norah S Al Shareef, Ali A AlThagafi, Tawfeeq S Sarraj, Ahmed Alnajrani

**Affiliations:** 1 Orthopedic Surgery, East Jeddah General Hospital, Jeddah, SAU; 2 Orthopedic Surgery, Al-Noor Specialist Hospital, Makkah, SAU; 3 Neurosurgery, Royal College of Surgeons in Ireland, Dublin, IRL; 4 Medical Oncology, University of Galway, Galway, IRL; 5 Medicine and Surgery, King Saud University, Riyadh, SAU; 6 Orthopedics, King Fahad General Hospital, Jeddah, SAU; 7 Orthopedics, Al Noor Hospital, Makkah, SAU; 8 Orthopedics, East Jeddah Hospital, Jeddah, SAU

**Keywords:** lateral ankle ligament instability, arthroscopic repair, complication, brostrom technique, ankle and foot

## Abstract

Injury to the lateral ligament is the most common cause of chronic lateral ankle instability. Lateral ankle instability is usually managed through conservative management, but surgery is indicated if this fails to relieve the symptoms. Surgical repair of the lateral ligament involves many surgical techniques including the arthroscopic repair technique and the modified Brostrom-Gould technique. Due to the minimal research on the complication rates of both techniques, this systematic review aims to establish the complication rates. To obtain articles, a detailed systematic search of MEDLINE, PubMed, Embase, Web of Science, and Cochrane Library was performed. The articles found using the keywords “arthroscopic,” “Brostrom,” and “Brostrom-Gould” were reviewed by two independent authors. The authors then selected the articles according to our predetermined eligibility criteria. The articles that met our inclusion were then chosen for data extraction. Specific details obtained from the study included the author’s details, the setting of the study, and the complications of the study. The online search yielded 975 articles, but only 44 met our inclusion criteria and were included in the review. The total sample size for the review was 2041 patients, the modified Brostrom technique was performed on 760 patients while on the remaining 1281 patients, arthroscopic repair was performed. On the characteristics of the sample, the age of the samples ranged from eight years to 83 years, while the mean BMI ranged from 21.0 kg/m² to 25.3 kg/m². The various complication rates included superficial peroneal nerve injury (2.3% in arthroscopic Brostrom and 0.65% in the Brostrom-Gould), wound infections (1.3% in arthroscopic Brostrom and 1.8% in the Brostrom-Gould), persistent pain (1.5% in the arthroscopic Brostrom and 1.1% in the Brostrom-Gould), and lastly recurrent instability (0.31% in arthroscopic Brostrom and 3.0% in the Brostrom-Gould). Overall, the complication rates of the arthroscopic repair were 11.00%, while those of the modified Brostrom-Gould were 10.65%. The study demonstrated that although the arthroscopic technique had higher complication rates than the modified Brostrom technique, the difference was insignificant. Therefore, we concluded that surgeons performing the arthroscopic Brostrom technique should have good arthroscopic skills to minimize complications.

## Introduction and background

Inversion stress to the foot and ankle is a common musculoskeletal injury, especially in athletes who are competing in various levels of sports. The ligaments most frequently damaged are the lateral ankle ligaments, specifically the calcaneofibular ligament and the anterior talofibular ligament [1]. These injuries are among the common causes of ankle instability in athletes. Evidence from research indicates that the rate at which it occurs in both men and women is almost equal [2]. The resulting chronic ankle instability can be classified into mechanical and functional instability. Functional instability is subjective according to the patient and is captured during history taking, while mechanical instability is objective and captured by the clinician during a physical exam [3].

The treatment modalities for chronic ankle instability include conservative management and physical therapy, and failure of both indicates surgery [3]. Conservative management is recommended in patients with symptoms for at least two months [3]. Conservative management involves the standard RICE (rest, ice, compression, and elevation) principles used to manage soft tissue injuries. Additionally, lateral heel wedges, proprioceptive training, peroneal strengthening, bracing and strapping, and lateral heel wedges are among the modalities used in ankle rehabilitation. Achilles tendon stretching and lateral heel wedges prevent hindfoot malpositioning, frequently leaving the lateral ligaments prone to injury [4]. On the other hand, peroneal strengthening and proprioceptive training stabilize the ankle and the hindfoot by improving the maintenance of ankle position when external forces are applied [4-6]. Ankle braces compensate for the instability, but those that provide clinically adequate stability tend to be bulky; hence, they are not routinely used [7]. Rehabilitation exercise forms a critical part of conservative management. They are continued for at least two to three months [8].

Surgery is usually carried out when conservative management fails to provide symptomatic relief. Surgical management is recommended after the patients have not improved after a three to six-month conservative management trial [9]. Various surgical techniques are used to manage chronic instability of the ankle. The methods range from simple repairs to complex reconstructions. The repairs include reattaching or shortening (imbricating) the damaged tissue. Reconstructions, on the other hand, include the replacement of ligaments with graft tissue, either allograft or autologous graft [9]. The reconstructive surgical techniques have evolved from the non-anatomical ones, e.g., Watson-Jones, Castaing, and Snook procedures, to the anatomical ones. Such developments have been due to possible complications of future ankle degeneration due to the non-anatomical forces associated with the non-anatomical techniques. Brostrom proposed the Brostrom technique in 1966 to avoid muscle imbalance by repairing the anterior talofibular ligament (ATLF) using the remnants [10]. Gould further modified this technique in 1980 by reinforcing the extensor retinaculum.

The modified Brostrom-Gould technique is primarily indicated for chronic, recurrent, lateral ankle instability. The method is only performed after the clients have undergone an entire course of physical therapy and failed to improve their symptoms [11]. Preoperative radiological imaging includes magnetic resonance imaging (MRI), which aids in assessing occult pathologies, including peroneal tendon and osteochondral pathologies. Additionally, a computed tomography (CT) scan can be done to get a complete evaluation of the pathology [12].

Hawkins pioneered the arthroscopic technique, which involved repairing the lateral ankle ligament with his stapling approach [13]. The method required two additional portals and ablation of the lateral surface before ligament plication with a staple. The series of 24 patients treated with the process had few complications. These complications were due to the placement of the staple. Kashuk et al. (1994) later developed a suture anchor technique that avoided the difficulties caused by prominent staples [14]. They used an additional accessory anterolateral portal to place suture anchors in the talus or the fibula [14]. Corte-Real and Moreira (2009) later attempted to develop a more reliable arthroscopic ligament repair. Their technique required an accessory anterolateral portal and used only one anchor. The technique had encouraging results as it had only two recurrences. However, the multiple suture passes contributed to slightly increased superficial peroneal nerve (SPN) injury (n = 3) [15]. In 2007, in potentially trying to avoid SPN injury, Acevedo and Mangeno (2015) started using another technique in which they tried to simplify all the prior methods. They used only two standard anterior portals. The technique allowed the placement of two individual suture anchors, which were believed to improve pull-out strengths and stability. Separate passes of sutures engaged a wider surface area, and Acevedo and Mangeno (2015) believed that this potentially avoided potential injury to the SPN [11].

The Brostrom-Gould technique and arthroscopic repair are the two main techniques used to repair lateral ankle injuries. Both techniques have shown effectiveness in restoring ankle stability. However, it is essential to understand the relative safety and occurrence of complications associated with each technique. The complications of the arthroscopic technique include SPN injury, recurrent instability, and unidentified pathology that continues to limit function and cause symptoms [12]. Additionally, the complications of the arthroscopic technique include persistent pain symptoms and postoperative neuritis. The complications' treatment included removing sutures that had trapped the SPN to treat the postoperative neuritis [11]. The postoperative neuritis was self-limiting for other patients, while others had chronic mild postoperative neuritis [11]. The complications of the Brostrom-Gould technique include persistent ankle pain laterally, subtalar laxity when the ankle is dorsiflexed, and wound complications a few weeks postoperatively. Oral antibiotics can treat the complications, or if the infection is severe, surgical debridement may need to be carried out [16].

Based on our knowledge, this is the first systematic review to directly analyze the complication rates of the Brostrom and Gould technique. The primary objective of this study is to compare the complication rates in patients undergoing arthroscopic repair of the lateral ankle ligament and the modified Brostrom-Gould technique. Specifically, the research aims to evaluate the overall complication rates associated with each technique; assess specific complications such as infection, wound healing issues, nerve damage, blood vessel injury, persistent pain, joint stiffness, and the need for additional surgeries; identify potential factors that may contribute to variations in complication rates between the two techniques; and lastly, establish various ways the complications are managed and if there are permanent sequelae of the various techniques.

## Review

Methodology

Study Design

This systematic review was prepared by observing and following the Cochrane Collaboration guidelines. The results were then reported according to the Preferred Reporting Items for Systematic Reviews and Meta-analyses (PRISMA) guidelines. The protocol used was not recorded in any database.

Eligibility Criteria

Two independent reviewers individually reviewed all the articles retrieved from the electronic databases. This was done according to our pre-determined eligibility criteria, which had inclusion and exclusion criteria. Studies were then selected in the review if they met the following inclusion criteria: (1) studies that compared arthroscopic versus modified Brostrom-Gould technique for lateral ankle ligament repair; (2) studies that reported clinical outcomes such as complications and treatment outcomes; (3) studies that included patients with lateral ankle repair; (4) studies that were randomized controlled trials (RCTs), quasi-randomized trials, any primary study, or prospective observational studies; (5) studies that are published in English to minimize translation of scientific words.

The studies were excluded from the review according to the following exclusion criteria: (1) studies that did not report the clinical outcomes; (2) studies that were not published in English; this criterion was essential in avoiding distortion of meaning for scientific terms' translation; (3) articles that were abstracts and lacking the full articles (non-full text), other meta-analyses, or systematic reviews.

Literature Search

Two authors independently conducted a thorough and systematic search for original and peer-reviewed articles. The authors implemented two strategies in their search: the first strategy involved using an outline search criterion on five electronic databases, which included MEDLINE, PubMed, Embase, Web of Science, and Cochrane Library. The search was limited to studies published in English up to July 2023. The search strategy used a combination of keywords related to the population, intervention, comparison, and outcome (PICO) of interest. The keyword for the population included “lateral ankle ligament.” The keywords for the intervention included “repair,” “open,” “arthroscopic,” “Brostrom,” and “Brostrom-Gould.” The outcomes of interest included “complications,” “treatment,” and “outcome.” The search terms were combined using the Boolean operators “AND,” “OR,” and “NOT,” in various databases as provided in Table 1.

**Table 1:** Search terms

The search terms were combined using Boolean operators "AND," "OR," and "NOT."	
Database	Search terms
PubMed/Embase/Web of Science/Ovid	“lateral” OR “ankle” OR “ligament” AND "Brostrom-Gould" OR “Brostrom” "repair" OR "repairing" OR "repairs" OR "open” OR "arthroscoped” OR "arthroscopes" OR "arthroscopes" OR "arthroscope” OR "arthroscopic” OR "arthroscopical” OR "arthroscopically” AND "complicances” OR "complicate” OR "complicated" OR "complicates” OR "complicating" OR "complication” OR "complications" OR “ outcome” "outcomes" “therapeutics” OR "therapeutics" OR "treatments” OR "therapy" OR "therapy” OR "treatment” OR "treatments"

After the eligible studies were obtained, the authors implemented the second strategy. This strategy involved manually searching the lists of references for other eligible studies that had not been identified through the electronic search. This enabled the authors to obtain all the possible articles. Any discrepancies were resolved through discussion by the reviewers and a third reviewer.

Quality Appraisal

Two independent reviewers evaluated the quality of the studies using the Newcastle-Ottawa quality assessment scale (NOS). A study was graded good quality if it had a total rating of 6 or more, fair quality if it had a 3 to 5 rating, and poor quality if it had a 2 or less rating. The results are shown in Table 2.

**Table 2:** Quality assessment outcomes of the included studies AHRQ: Agency for Healthcare Research and Quality.

Author	Selection	Comparability	Outcome	AHRQ standard
Letts et al. (2003) [17]	1	0	2	Fair
Li et al. (2022) [18]	1	0	2	Fair
Li et al. (2017) [19]	2	0	3	Fair
Liu et al. (2023) [20]	2	0	3	Fair
Lui (2021) [21]	1	0	2	Fair
Lopes et al. (2018) [22]	3	1	3	Good
Maffulli et al. (2013) [23]	2	0	3	Fair
Matsui et al. (2016) [24]	2	1	3	Good
Mederake et al. (2022) [25]	2	1	3	Good
Molloy et al. (2014) [26]	2	0	3	Fair
Nery et al. (2011) [27]	2	0	3	Fair
Petrera et al. (2014) [28]	2	0	3	Fair
Rigby and Cottom (2019) [29]	2	0	3	Fair
Tan et al. (2023) [30]	2	0	3	Fair
Vega et al. (2020) [31]	2	0	3	Fair
Ventura et al. (2022) [32]	2	1	3	Good
Wang et al. (2023) [33]	2	0	3	Fair
Wang et al. (2023) [34]	2	1	3	Good
Wei et al. (2019) [35]	2	0	3	Fair
Xu et al. (2020) [36]	2	0	3	Fair
Xu and Lee (2016) [37]	1	0	3	Fair
Zeng et al. (2020) [38]	2	0	3	Fair
Zhang et al. (2020) [39]	2	0	3	Fair
Zhou et al. (2021) [40]	2	1	3	Good
Keller et al. (1996) [41]	2	0	3	Fair
Corte-Real and Moreira (2009) [15]	2	0	3	Fair
Kim et al. (2010) [42]	2	0	3	Fair
Lee et al. (2011) [43]	2	0	3	Fair
Buerer et al. (2013) [44]	2	0	3	Fair
Brodsky et al. (2005) [45]	2	0	3	Fair
Ferkel and Charms (2007) [46]	2	0	3	Fair
Acevedo and Mangone (2011) [12]	2	0	3	Fair
Labib and Slone (2015) [47]	2	0	2	Fair
Kocher et al. (2017) [16]	2	0	3	Fair
Chew et al. (2018) [48]	2	0	3	Fair
Hassan et al. (2018) [49]	2	0	3	Fair
Lee et al. (2019) [50]	2	0	3	Fair
Ming et al. (2020) [51]	2	0	3	Fair
Hagio et al. (2020) [52]	2	0	3	Fair
Kanzaki et al. (2020) [53]	2	0	3	Fair
Baek et al. (2023) [54]	2	1	3	Good
Hou et al. (2022) [55]	2	0	3	Fair
Kim et al. (2019) [56]	2	0	3	Fair
Alnejadi et al. (2023) [57]	2	0	3	Fair

Data Extraction and Evaluated Outcomes

The articles that met the inclusion criteria were independently reviewed by two reviewers. The reviewers obtained the relevant data for review. The specific data that were collected from each study included the first author's maiden name and publication year, the study design, the characteristics of the participants (mean age and mean BMI), the type of surgical technique used, the sample size, the mean follow-up period, and the primary outcomes. The primary outcomes included were the complications of the various techniques used.

Results

**Figure 1:**
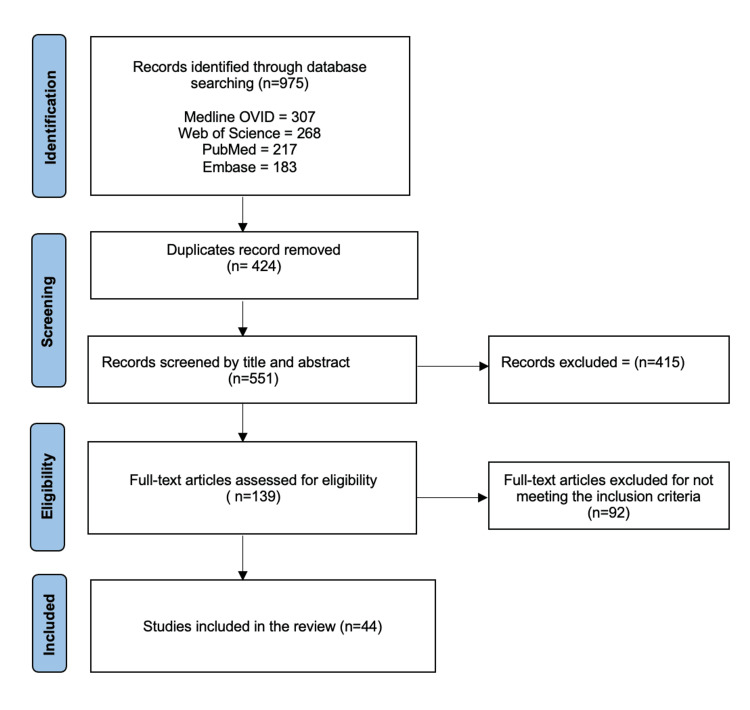
PRISMA flow diagram PRISMA: Preferred Reporting Items for Systematic Reviews and Meta-Analyses.

The online search conducted through various electronic databases stated earlier yielded 975 articles. After careful analysis of these articles, 424 duplicates were identified, which the reviewer excluded from the study. The reviewers then screened the remaining 551 articles’ abstracts and titles; all 551 met the screening criteria, and the reviewers could not retrieve 415 articles. The remaining 139 were now screened using the pre-determined eligibility criteria. After the assessment, 40 articles were excluded because they were not published in English, 48 articles were reviews and meta-analyses, hence excluded, and two others were excluded because they were letters to the editor. The final articles to be included were 44, which met all our inclusion criteria. The results of the search are shown using the PRISMA flow diagram (Figure 1).

Various surgical techniques were used in the studies, including the arthroscopic Brostrom technique and the modified Brostrom-Gould technique. Only the results of the complication rates of the arthroscopic Brostrom technique and modified Brostrom-Gould technique were included in comparative studies where various methods were used in lateral ankle repair. The study's total patient sample had 2041 patients; 760 patients underwent the modified Brostrom-Gould procedure, while the remaining 1281 patients underwent arthroscopic Brostrom repair. The complication rates varied from no complication rates in some case studies and case reports to as high as 27% complication rates in some studies. The complications of the various techniques included superficial wound infection, SPN and sural nerve injuries, knot pain, recurrent instability of the ankles, persistent pain, neuromas, and suture knot granulomas, among others. The various complication rates for the two techniques are displayed in Table 3.

**Table 3:** The complication rates of the two surgical techniques

Complication	Arthroscopic Brostrom	Modified Brostrom-Gould
Superficial peroneal nerve injury	2.3%	0.65%
Wound infection	1.3%	1.8%
Persistent pain	1.5%	1.1%
Recurrent instability	0.31%	3.0%
Other complications	5.59%	4.1%
Overall complication rate	11.00%	10.65%

The characteristics of the various studies have been displayed in Table 4.

**Table 4:** Study characteristics AB: arthroscopic Brostrom-Gould technique; OB: open Broström-Gould technique; AS group: arthroscopic Broström–Gould repair group; SPN: superficial peroneal nerve; AOFAS: American Orthopaedic Foot and Ankle Society.

Author	Country	Type of study	Study characteristics	Sample size	Surgical technique	Follow-up time	Primary outcomes
Letts et al. (2003) [17]	Canada	Retrospective review	Average age = 14 years and three months	12	Brostrom technique	Average 3 years 1 month	Only 1 child (8.3%) had an unstable ankle after treatment.
Li et al. (2022) [18]	China	Retrospective review	Mean age = 30.17 years	29	Arthroscopic repair	18.66 ± 4.85 months	8 patients (27.5%) had malleolar avulsion fractures, and 10 had talus cartilage injury.
Li et al. (2017) [19]	China	Cohort study	Mean age = 30.3 ± 10.1 years, mean BMI = 23.3 ± 2.9 kg/m²	6l	Arthroscopic repair	2 years	One patient (4.3%) had no improvement in the functional scores due to an osteochondral defect in the medial talus.
Liu et al. (2023) [20]	China	Retrospective review	Mean age = 35 (16-60) years	39	Arthroscopic Brostrom	22 months	2 patients (5.1%) had superficial peroneal nerve irritation symptoms. 3 patients (7.7%) had mild pain anteroinferior to the lateral ankle.
Lui (2021) [21]	China	Case report		1	Arthroscopic Brostrom	2 years	The patient had no complications.
Lopes et al. (2018) [22]	France	Prospective cohort	Mean age = 32.4 (12-67) years, mean BMI = 24.9 (18-52) kg/m²	286	Arthroscopic repair	9.6 months	4.2% of patients had infectious complications, 10% had neurological complications, and 3.5% had a neuroma.
Maffulli et al. (2013) [23]	United Kingdom	Case series		38	Ankle arthroscopy and Brostrom repair	8.7 years	11% (4) of patients developed surgical complications. 1 (3%) had a superficial wound infection that healed. 3 had sensory disturbance of the lateral foot aspect, which was self-limiting.
Matsui et al. (2016) [24]	Japan	Retrospective cohort	Mean age = 28 years (8-59 years)	37	Arthroscopic repair	1 year	2 (5.4%) patients had temporary numbness of SPN, while 3 (8.1%) had wound irritation.
Mederake et al. (2022) [25]	Germany	Retrospective cohort	Median age = 35 years (17-54) years	14	Arthroscopic Brostrom	1 year	14% of patients had complications. 1 (7%) had a suture granuloma, while 1 (7%) had nerve damage.
Molloy et al. (2014) [26]	United Kingdom	Prospective cohort	Mean age = 33 (20-46 years)	18	Modified Brostrom-Gould	25 months	One patient (5.6%) had scar tenderness due to a palpable suture.
Nery et al. (2011) [27]	United Kingdom	Case series	Mean age = 28.8 years	38	Brostrom-Gould	9.8 years	2 patients (5.3%) had low AOFAS scores.
Petrera et al. (2014) [28]	Canada	Cohort study	Mean age = 25 (18-37) years	55	Modified Brostrom repair	42 months	There was a 6% failure rate. 3 (5.5%) patients had a traumatic retear that resulted in residual instability. 2 (3.6%) patients had superficial wound infection. Temporary neurapraxia of SPN was reported in one of the patients.
Rigby and Cottom (2019) [29]	United States	Retrospective study	Mean age = 47.89 (14-83) years	62	Modified Brostrom-Gould	3.7 years	2 patients (6.5%) reported superficial peroneal neuritis.
Tan et al. (2023) [30]	Singapore	Retrospective study	Mean age = 34.6 (19-61) years	42	Modified Brostrom-Gould surgery	2.6 years	2 patients (4.8%) suffered exercise-induced ankle sprains.
Vega et al. (2020) [31]	Spain	Cohort study	Median age = 41 (22-56) years	24	Arthroscopic all-in-repair	35 months	2 (8.3%) patients had symptomatic arthrofibrosis of the anterior ankle. 1 patient (4.2%) had SPN neuralgia and numbness. 2 patients had delayed anteromedial portal healing. 1 patient (4.2%) complained of discomfort in the lateral ankle.
Ventura et al. (2022) [32]	Italy	Retrospective study	The study did not provide sample characteristics	18	Arthroscopic Brostrom technique	4 years	2 (11%) patients had complications. The complications were SPN injury in 1 case (5.5%) and another (5.5%) of persistent pain near a suture knot.
Wang et al. (2023) [33]	China	Prospective cohort	In the arthroscopic group, the mean age was 28.6 ± 8.1 years, and the mean BMI was 22.9 ± 5.1 kg/m²; in the open group, the mean age was 27.2 ± 7.7 years and the mean BMI was 22.9 ± 5.1 kg/m²	32 arthroscopic, 32 Brostrom-Gould	Arthroscopic Brostrom and open ± Brostrom-Gould	11.25 months	1 patient (3.2%) in the open group and 2 patients (6.4%) in the Brostrom-Gould had dorsiflexion range of motion (ROM) restriction.
Wang et al. (2023) [34]	China	Retrospective review	The mean age of the patients of AB was 31.71 ± 4.99 and OB was 31.92 ± 4.77. The mean BMI for AB was 21.84 ± 2.40 kg/m² while for OB was 22.38 ± 2.29 kg/m²	AB = 49 and OB = 50	Arthroscopic Brostrom and Brostrom-Gould method	48 months	Arthroscopic Brostrom had higher complications (16.3%) compared to the Brostrom-Gould technique (14%). Arthroscopic Brostrom had 8 patients; 6 patients (12.2%) had SPN injury, and 2 (4.1%) had superficial wound infection. In Brostrom-Gould, 7 patients had complications: 2 had SPN injuries and 5 had superficial wound infections.
Wei et al. (2019) [35]	China	Cohort study	The average age was 34.3 ± 10.3 years, and 11 were smokers	29 patients	All inside arthroscopic repair	33.7 months	There were only 3 cases (10.3%) of irritation of the suture knot site. 2 (6.9%) patients had an additional ankle sprain.
Xu et al. (2020) [36]	China	Retrospective study	Age = 33.7 ± 7.0 years; BMI = 23.3 ± 2.3	32 patients	All arthroscopic repair	24 months	3 patients (9.4%) had SPN injury, while an additional 2 patients (6.2%) had knot pain.
Xu and Lee (2016) [37]	Korea	Cohort study	52 were below 33 years, while 48 were above 33 years. 54 had a BMI of less than 24, while 46 had a BMI above 24	100 patients	Modified Brostrom procedure	43.3 months	There were 11.4% major complications, which were ruptures and recurrent lateral ankle instability complication rates in the laxity group and 1.8% in the non-laxity group. 5 cases of failure were reported in the laxity group, i.e., Beighton scores < 9. The rate of minor complications was 6.8% (3) and 8.9% (5 patients) in the laxity and non-laxity groups, respectively. These included superficial infections, ankle range of motion loss, and sural nerve irritation. Four patients had reduced sagittal ankle plane motion. 3 patients had superficial wound infections, and 1 had a sural nerve injury.
Zeng et al. (2020) [38]	China	Retrospective cohort	Mean age = 29.7 ± 7.6 years, mean weight = 69.9 ± 11.2 kg	27 patients, 10 = OB, 17 = AB	Open Brostrom technique and arthroscopic surgery	3 years	In the open group, there was poor healing in 2 patients (20%) and a painful nodule in 1 patient (10%). In the arthroscopic group, there was 1 (5.9%) patient with poor healing and 1 patient (5.9%) with nerve injury.
Zhang et al. (2020) [39]	China	Retrospective cohort	Mean age = 32.4 ± 2.4 years, BMI = 24.6 ± 3.4	28	Arthroscopic reconstruction	24 months	2 patients (7.14%) had SPN injury and 1 patient (3.6%) had sural nerve injury. There was additional skin irritation in 2 patients.
Zhou et al. (2021) [40]	China	Retrospective cohort	Mean age = 33.42 ± 6.40 years, mean BMI = 24.42 ± 1.87.	31 patients	Arthroscopic repair and modified Brostrom-Gould	2 years	In the arthroscopic group, complications included 1 case (3.23%) of SPN neuritis, and 1 patient (3.23%) had recurrent instability. In the open Gould technique, the complications included 2 patients (5.56%) who had recurrent instability, 1 patient (2.78%) who had lateral ankle sensory disturbance, and 1 patient (2.78%) who had mild skin irritation.
Keller et al. (1996) [41]	United States of America	Clinical study	Mean age = 33.8.	44 patients	Brostrom-Gould procedure	2.6 years	Two patients (4.5%) had small, uncomplicated wound dehiscence at the apex of the incision, 6 patients (13.66%) had edema, and 1 patient (2.3%) had continued pain, stiffness, and recurrent sprains.
Corte-Real and Moreira (2009) [15]	Portugal	Retrospective case study	Not provided	28 patients	Arthroscopic repair	24.5 months	9 patients (29%) had complications, 3 (9.6%) had persistent problems. 2 (7.2%) patients had delayed wound healing, 3 (9.6%) had SPN numbness, and 1 (3.6%) had deep venous thrombosis.
Kim et al. (2010) [42]	Korea	Retrospective comparative study	Mean age = 26.2 ± 9.1 ossicle group, 28.4 ± 10.4	69 patients	Modified Brostrom technique	47 months	2 patients (7.7%) had recurrent ankle sprains; in another group, two patients (7.7%) had recurrent instability.
Lee et al. (2011) [43]	Korea	Retrospective case series	Mean age = 23.0 ± 5.3	30 patients	Modified Brostrom	10.6 years	They did not report any complications.
Buerer et al. (2013) [44]	Switzerland	Retrospective cohort	Mean age = 33.7(18-60) years, mean BMI = 24.69 kg/m² (18.99-33.46 kg/m²)	41 patients	Modified Brostrom-Gould procedure	13-72 months	3 patients (7.3%) had complications, 1 (2.4%) had perimalleolar pain, 1 (2.4%) had persistent numbness of the ankle, and 1 (2.4%) had pain and discomfort in the area of the scar.
Brodsky et al. (2005) [45]	USA	Retrospective	Mean age = 31 (15-61) years	73 patients	Modified Brostrom-Gould procedure	64 months	1 patient (1.4%) had a superficial ulcer on the fifth metatarsal head, 1 (1.4%) had reflux sympathetic dystrophy, and 1 (1.4%) had tibial sesamoiditis.
Ferkel and Charms (2007) [46]	USA	Retrospective	Mean age = 28 (13-61) years	21 patients	Modified Brostrom-Gould	60 months	3 patients (14.3%) had traumatic sprains after surgery.
Acevedo and Mangone (2011) [12]	USA	Retrospective	Average age = 39 (15-55) years	23	Arthroscopic lateral ankle reconstruction	10.9 months	1 patient (4.3%) had a neurological problem and proximal muscle weakness, 1 patient (4.3%) had peroneal tendonitis, and 1 patient (4.3%) had sural nerve neuritis.
Labib and Slone (2015) [47]	USA	Retrospective	Not provided	14 patients	Arthroscopic Brostrom-Gould repair	3 months	2 patients (14.2%) were still experiencing some pain.
Kocher et al. (2017) [16]	USA	Retrospective	Mean age = 14.9 ± 2.7 years	31 patients	Brostrom procedure	36.2 months	1 patient (3.2%) had persistent pain; 2 patients (6%) had wound complications.
Chew et al. (2018) [48]	Singapore	Retrospective	Mean age = 24.13 (range = 18-42) years	24	Modified Brostrom-Gould procedure	28.7 months	The study did not report any complications.
Hassan et al. (2018) [49]	UK	Retrospective	Mean age = 30.1 (range 15.8-47.4) years	27	Modified Brostrom technique	5 years	1 patient (3.7%) complained of pain at the site of the scar.
Lee et al. (2019) [50]	Korea	Case report	34 years	1	Arthroscopic modified Brostrom technique	6 months	The patient had severe pain on the lateral aspect of the right ankle.
Ming et al. (2020) [51]	China	Retrospective	Mean age = 32.17 ± 6.35 years, mean BMI = 22.69 ± 5.13 kg/m²	37 patients	Arthroscopic anatomical repair	33.16% ± 10.58 months	The study did not have any complications.
Hagio et al. (2020) [52]	Japan	Retrospective comparative study	Mean age = 28 (12-66) years	47 patients	Arthroscopic lateral ankle repair	14 months	The study did not report any complications.
Kanzaki et al. (2020) [53]	Japan	Case series	Mean age = 30.0 years	47 patients	Arthroscopic lateral ankle repair	21 months	1 patient (2.1%) had residual joint pain due to residual osteophytes, 2 patients (4.2%) had scar pain, and 1 patient (2.1%) had SPN irritation and a positive Tinel sign.
Baek et al. (2023) [54]	Korea	Retrospective case cohort	Mean age = 22.0 ± 4.0 years. The mean BMI in the AS group was 25.2 ± 3.6 kg/m² and in the open group was 24.1 ± 3.6 kg/m²	31 patients in the AS group, 34 patients in the open group	Arthroscopic Brostrom-Gould technique and open Brostrom-Gould repair	50.8 months	One patient (3.2%) in the AS group had transient damage to the SPN.
Hou et al. (2022) [55]	China	Randomized controlled trial	Mean age = 28.3 ± 5.4, mean BMI = 21.0 ± 3.1	36 patients	Arthroscopic Brostrom procedure	2 years	One patient (2.8%) complained of joint instability.
Kim et al. (2019) [56]	Korea	Retrospective comparative study	Mean age = 36.1 ± 14.5 years	133 patients	Arthroscopic modified Brostrom procedure	12 months	2 patients (1.6%) developed SPN injury, 1 patient (0.8%) had knot pain, and 1 (0.8%) had an abscess.
Alnejadi et al. (2023) [57]	Saudi Arabia	Case series	Mean age = 36 years, mean BMI = 37.7 kg/m²	8 patients	Modified Brostrom technique	1 year	2 patients (25%) had persistent pain. After 1 year of follow-up.

The characteristics of the sample populations included the mean age and mean body mass index (BMI) where one was provided. The minimum age in the included studies was eight years, and the maximum age was 83 years. The mean BMI ranged from 21.0 kg/m² to 25.3 kg/m². One study provided the mean weight, which was 69.9 kg. The mean BMI ranged from 21.0 kg/m² to 25.3 kg/m².

Discussion

This systematic review discussed the complication rates of various studies on the arthroscopic Brostrom technique and the modified Brostrom-Gould technique. The most important finding in the arthroscopic Brostrom technique was that complication rates ranged from 0% in some studies to 29% in other studies [15,51,52]. For the modified Brostrom-Gould technique, on the other hand, the complication rates ranged from 0% complication rates in some studies to 30% complication rates in others [38,48]. The absolute mean complication rate of the studies reviewed for the modified Brostrom-Gould procedure is 10.65%, while the one for the arthroscopic repair is 11.00%. The complication rates in the arthroscopic Brostrom repair were higher than those of modified Brostrom-Gould but not significantly. These results are similar to those of a systematic review by Brown et al. (2018), who reported higher complication rates in the arthroscopic Brostrom technique (11.5%) compared to 5.4% in the Brostrom-Gould technique [58]. Additionally, the rates of complications of arthroscopic Brostrom procedure are similar in both reviews. However, the rates of complication in the modified Brostrom-Gould technique in our systematic review (10.65%) are slightly higher compared to those of Brown et al. (2018) (5.4%), and this may be attributed to our large sample size, which improved the statistical ability of our review [58].

Regarding specific complications, cases of SPN injury and neuritis were reported among various studies and across all the techniques. The rate of SPN injury in modified Brostrom techniques was minimal at a rate of 0.65%, while that of the arthroscopic Brostrom repair was higher with the rate of SPN injury at 2.3% of the sample of patient’s studies. The higher complication rates of SPN injury associated with the arthroscopic Brostrom technique are similar to those reported by Wang et al. (2014), who reported that SPN injuries are frequently reported complication in the studies they reviewed [59]. To aid in minimizing this risk, Acevedo and Mangone (2015) further recommended that the orthopedic surgeons performing the procedure must possess a profound knowledge of the ankle and foot anatomy and have excellent arthroscopic skills [11]. Vega et al. (2013) further add that surgeons should be cautious when creating portals, inserting the instruments, and carrying out the procedure, as this minimizes the risk of nerve injuries [60].

Infectious complications postoperatively are common. In both groups, wound infection and healing difficulties were reported. The rates of infectious complications in the arthroscopic group were 1.3%, while those in the modified Brostrom group were 1.8%. Most infections were self-limiting, while others had to be treated with antibiotics. The pathogens observed to cause the infections included pseudomonas and Peptostreptococcus species, seen in wound cultures [16]. The treatment regimen consisted of clindamycin or cephalexin. In severe cases, some wounds had to undergo irrigation and surgical debridement. All the cases of wound infection reported were successfully managed, and residual infection existed.

The arthroscopic repair was initially developed as a minimally invasive procedure, and its use has become popular as it reduces recovery pain and postoperative pain. Another complication reported was postsurgical pain. The pain ranged from one caused by nerve irritation to another caused by knot irritation. The rates of surgical pain caused included 1.1% in the Brostrom-Gould group and 1.5% in the arthroscopic Brostrom technique. Some of the pain was self-limiting and subsided on its own, some were treated with nonsteroidal anti-inflammatory drugs while other types became recurrent and persistent [40].

Other complications were recurrent instabilities after the procedures. The rate of recurrent instability was lower in the arthroscopic Brostrom repair (0.31%) compared to the modified Brostrom-Gould technique (3.0%). The rate of recurrent instability of the modified Brostrom technique is less than that reported by Lewis et al. (2021), i.e., 9.28% [61]. The authors attributed these phenomena to the patients resuming their activities, as most of the injuries were a result of postoperative injuries. The patients were placed on conservative management without the need for revision surgery [28]. Additionally, Zhou et al. (2021) recommended that they wear ankle pads during exercises for additional protection. The instability did not provide a significant disturbance in their activities of daily living [40].

Limitations of the study

One of the limitations of this study is that some of the included studies have small sample sizes. The drawback contributed to the small sample size for the specific complications of the various techniques. Although our sample size and included studies are slightly larger than the one for other systematic reviews, the specific sample sizes of the various individual complications were limited, and we would recommend that future studies focus on the specific complications and assess their rates for each of the procedures. Another limitation was on the characteristics of the included populations; hence, we could obtain only the mean age for the various studies. This reduced our ability to relate the complication rates to the sample populations' various characteristics.

## Conclusions

This systematic review established that the rate of complications is higher in the arthroscopic repair compared to the modified Brostrom-Gould procedure. The wound infection rate was, however, higher in the modified Brostrom-Gould group than in the arthroscopic group. The rate of injuries to the SPN was higher in the arthroscopic group compared to the Brostrom-Gould group; hence, we recommended that caution be observed by surgeons who are operating and also the surgeons who possess good arthroscopic skills before performing the procedure. In summary, the difference between the complications of the Brostrom-Gould and arthroscopic repair procedures was insignificant; hence, we can conclude that both procedures are productive in avoiding complications. We recommend that future systematic reviews be conducted to compare the two techniques regarding specific complications.
